# Protocol of a randomized open label multicentre trial comparing continuous intrajejunal levodopa infusion with deep brain stimulation in Parkinson’s disease - the INfusion VErsus STimulation (INVEST) study

**DOI:** 10.1186/s12883-020-1621-y

**Published:** 2020-01-31

**Authors:** D. van Poppelen, V. Sisodia, R. J. de Haan, M. G. W. Dijkgraaf, P. R. Schuurman, G. J. Geurtsen, A. E. M. Berk, R. M. A. de Bie, J. M. Dijk

**Affiliations:** 1 Department of Neurology, Amsterdam UMC, University of Amsterdam, Meibergdreef 9, Amsterdam, the Netherlands; 2 Clinical Research Unit, Amsterdam UMC, University of Amsterdam, Meibergdreef 9, Amsterdam, the Netherlands; 3 Department of Clinical Epidemiology, Biostatistics and Bioinformatics, Amsterdam UMC, University of Amsterdam, Meibergdreef 9, Amsterdam, the Netherlands; 4 Department of Neurosurgery, Amsterdam UMC, University of Amsterdam, Meibergdreef 9, Amsterdam, the Netherlands; 5 Department of Medical Psychology, Amsterdam UMC, University of Amsterdam, Meibergdreef 9, Amsterdam, the Netherlands; 6 Dutch Parkinson’s disease association (Parkinson Vereniging), Kosterijland 12, Bunnik, the Netherlands

**Keywords:** Parkinson’s disease, Deep brain stimulation, Continuous intrajejunal levodopa infusion, Cost-effectiveness analyses, Patient preference trial, Randomized controlled trial

## Abstract

**Background:**

Both Deep Brain Stimulation (DBS) and Continuous intrajejunal Levodopa Infusion (CLI) are effective therapies for the treatment of Parkinson’s disease (PD). To our knowledge, no direct head-to-head comparison of DBS and CLI has been performed, whilst the costs probably differ significantly. In the INfusion VErsus STimulation (INVEST) study, costs and effectiveness of DBS and CLI are compared in a randomized controlled trial (RCT) in patients with PD, to study whether higher costs of one of the therapies are justified by superiority of that treatment.

**Methods:**

A prospective open label multicentre RCT is being performed, with ancillary patient preference observational arms. Patients with PD who, despite optimal pharmacological treatment, have severe response fluctuations, bradykinesia, dyskinesias, or painful dystonia are eligible for inclusion. A total of 66 patients will be randomized. There is no minimal inclusion in the patient preference arms. The primary health economic outcomes are costs per unit on the Parkinson’s Disease Questionnaire-39 (PDQ-39) and costs per unit Quality-Adjusted Life Year (QALY) at 12 months. The main clinical outcome is patient-reported quality of life measured with the PDQ-39 at 12 months. Patients will additionally be followed during 36 months after initiation of the study treatment.

**Discussion:**

The INVEST trial directly compares the costs and effectiveness of the advanced therapies DBS and CLI.

**Trial registration:**

Dutch Trial Register identifier 4753, registered November 3rd, 2014; EudraCT number 2014–001501-32, Clinicaltrials.gov: NCT02480803.

## Background

Parkinson’s disease (PD) is a neurodegenerative disease affecting motor, autonomic, cognitive, and sensory systems. Several symptomatic therapies are available, with levodopa as the mainstay, which generally have a good effect upon the motor symptoms. Due to disease progression in conjunction with the pharmacokinetics of the dopaminergic medication, patients frequently develop rapid and seemingly unpredictable swings between mobility, often with dyskinesias (on phase), and immobility (off phase) [1]. For these patients, continuous electrical stimulation through Deep Brain Stimulation (DBS), continuous dopaminergic treatment with Continuous intrajejunal Levodopa Infusion (CLI), and Continuous subcutaneous Apomorphine Infusion (CAI) are available. Globally, the prevailing treatments for patients with advanced PD differ between countries, regions and neurologists. DBS seems to be the most established therapy, being available for over 25 years in some countries [2–4]. Several randomized clinical trials (RCTs) have shown that DBS is efficacious for the treatment of PD motor symptoms: it reduces motor fluctuations and dyskinesias [5]. CLI has also been shown to reduce daily off-time and is effective for the treatment of motor fluctuations and dyskinesias, as was shown in some RCTs that mostly included relatively few patients and had a short follow-up [6–9]. Both therapies significantly improve quality of life [5, 6, 10–14]. Regarding CAI, a recent randomized placebo-controlled trial showed a reduction in off-drug time and an increase in on-drug time without troublesome dyskinesia compared to placebo after three months follow-up, whereas quality of life did not differ [15].

Concerning the therapies DBS and CLI, both patients and neurologists tend to prefer CLI over the traditionally standard treatment DBS according to both surveys that were performed by us (unpublished) and surveys that were published [16]. This is of interest considering the relative lower level of evidence for the use of CLI, but also because CLI probably is substantially more expensive than DBS [17]. Whether or not such cost difference is justified by a difference in effectiveness is unknown as no head-to-head comparison of DBS and CLI has been performed. The INfusion VErsus STimulation (INVEST) study aims to directly compare costs and effectiveness of DBS and CLI in an RCT in patients with PD to study whether higher costs of one of the therapies are justified by superiority of that treatment. Additionally, motor and non-motor symptoms, daily functioning, quality of life and (serious) adverse events ((S)AE)s will be compared between the two therapies.

## Methods

### Study design

**Fig. 1:**
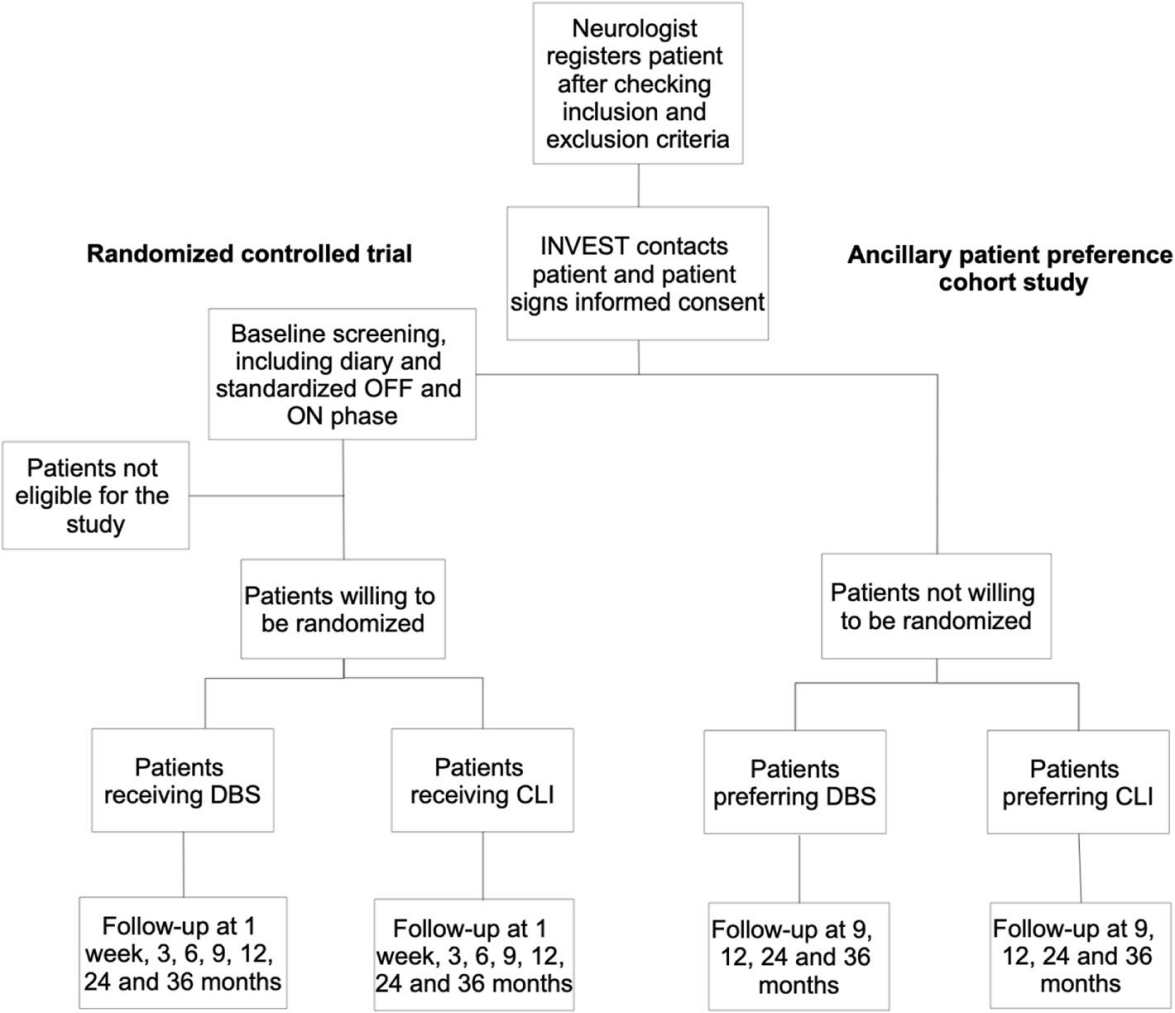
Flowchart. CLI, Continuous intrajejunal Levodopa Infusion; DBS, Deep Brain Stimulation

A prospective open label multicentre RCT will be performed. Patients who are eligible to participate in the RCT but do not want to be randomized, will be asked to participate in the ancillary patient preference observational study (Fig. 1). This design of an RCT with ancillary patient preference observational arms is known as a “patient preference trial” or “comprehensive cohort study” [18].

### Patients

Patients with advanced PD who are eligible for treatment with both DBS and CLI will be recruited from academic and non-academic hospitals. In the Netherlands, DBS treatment is carried out in 7 centres of which 4 are academic and CLI treatment is carried out in numerous mainly non-academic centres. Both therapies are unconditionally reimbursed by health insurers in the Netherlands. We aim for a study population that is illustrative of the current clinical practice in the Netherlands with patients who will be treated in a variety of centres according to usual care.

In order to be eligible to participate in this study, a subject must meet all of the following inclusion criteria: (a) age 18 years and older; (b) idiopathic PD diagnosed by their treating neurologist with bradykinesia and at least two of the following signs: resting tremor, rigidity, and asymmetry; (c) despite optimal pharmacological treatment, at least one of the following symptoms: severe response fluctuations, dyskinesias, painful dystonia or bradykinesia; and (d) a life expectancy of at least two years. Exclusion criteria are: (a) previous PD-neurosurgery (e.g., DBS, pallidotomy, thalamotomy); (b) contraindications for DBS surgery, such as a physical disorder making surgery hazardous; (c) previous CLI (through a Percutaneous Endoscopic Gastrostomy (PEG) tube or Nasal Jejunal tube); (d) contraindications for PEG surgery such as interposed organs, ascites and esophagogastric varices, or for CLI treatment; (e) Hoehn and Yahr stage 5 at the best moment during the day [19]; (f) other, severely disabling condition; (g) dementia or indication of severe cognitive impairment, such as Parkinson’s Disease-Cognitive Rating Scale (PD-CRS) < 65; (h) psychosis; (i) current depression; (j) pregnancy, breastfeeding, and women of childbearing age not using a reliable method of contraception; (k) legally incompetent adults and (l) no informed consent.

### Study procedures and randomization

Members of the INVEST research team will contact and inform eligible patients interested in the study after they have given their treating neurologist permission to forward contact details to the INVEST-team. Informed consent will be signed by patients willing to participate in the RCT or ancillary patient preference cohort study.

#### Randomized controlled trial

Patients willing to be randomized will firstly undergo an evaluation to assess eligibility for treatment with both DBS and CLI including (a) imaging of the brain (preferably MRI); (b) standardized motor evaluation using the Movement Disorder Society - Unified Parkinson’s Disease Rating Scale (MDS-UPDRS part III) in off medication and on medication phase; (c) neuropsychological testing; (d) psychiatric evaluation by means of questionnaires (Hamilton Anxiety Rating Scale (HAM-A), Hamilton Depression Rating Scale (HAM-D), selected items of the Mini-International Neuropsychiatric Interview version 5.0 (MINI; items: A: major depressive episode, major depressive disorder; D: (hypo)manic episode; E: panic disorder; F: agoraphobia; G: social anxiety disorder; H: obsessive-compulsive disorder; J: alcohol use disorder; K: substance use disorder; L: psychotic disorder), Columbia Suicide Severity Rating Scale) or through psychiatric consultation which is mandatory when the treating physician considers this indicated or when the results of the questionnaires indicate a potential contra-indication for treatment with DBS or CLI; (e) limited laboratory testing and (f) appraisal of potential contra-indications for treatment with DBS or CLI. The results of the evaluation will be discussed with the appropriate disciplines (neurology, neuropsychology, neurosurgery, and if considered necessary, gastroenterology) in the treating centre and eligibility for treatment with both DBS and CLI will be determined multi-disciplinary. In case of a contra-indication or ineligibility for (one of) the treatments, the patient will be excluded from the study and will not be randomized. Possible contra-indications are a tremor as main complaint that appears not responsive to dopaminergic medication according to the patient and during the standardized off-drug and on-drug evaluation, or if the effect of dopaminergic medication on motor symptoms is considered too small according to the treating physicians. Patients who are eligible for both therapies will subsequently be randomized using a web-based application, using randomly permuted blocks with block sizes 2, 4 and 6. Randomization will be stratified by level of experience of the treatment centre (which may not be the centre including the patient) in four strata (i.e. 1. inexperienced regarding both DBS and CLI treatment; 2. experienced in DBS but inexperienced in CLI; 3. experienced in CLI but inexperienced in DBS or 4. experienced in both DBS and CLI treatment). For this stratification, a centre is considered experienced in DBS or CLI treatment if in the previous two years at least 5 patients per year started with DBS or CLI respectively in that centre. If a center gains more experience in one of the therapies, the relevant status may change.

#### Ancillary patient preference cohort study

Patients who decide not to participate in the RCT, but are willing to take part in the ancillary patient preference cohort study, can either be registered by their treating neurologist, or can contact the investigators directly, after which the in- and exclusion criteria will be verified with their treating neurologist.

### Treatment procedures

The patient will receive the allocated treatment in the initial hospital or, if the specific treatment (i.e., DBS or CLI) is unavailable in that centre, in a nearby cooperating DBS or CLI centre according to agreements made prior to randomization. The allocated or preferred treatment will be carried out according to usual care and according to the custom treatment protocols of the treatment centre at that moment. For patients participating in the RCT, the study-treatment will be initiated within three months after the screening for eligibility.

#### Deep brain stimulation treatment

For DBS, the Leksell stereotactic frame will be employed to implant two electrodes, guided by Magnetic Resonance Imaging (MRI), under local or generalized anesthesia depending on local protocol. Bilaterally, a four-contact electrode (Medtronic, Minneapolis, MN, USA) will be implanted in the subthalamic nucleus (STN). Subsequently, the pulse generator will be subcutaneously implanted in the subclavian area under general anesthesia. The electrodes will be connected to the pulse generator. Patients are hospitalized for four days and do not receive PD drugs on the day of surgery until the end of the procedure. During the course of the study, the use of oral co-medication is allowed, as in regular daily practice and changes in drug treatment are allowed. Patients will regularly visit the outpatient clinic to adjust stimulation parameters and PD medication while assessing the interaction between both treatments. The treating neurologist supervises any changes in medication.

#### Continuous levodopa infusion

In CLI, a levodopa-gel is continuously administered through a tube in the jejunum (Duodopa, Abbott, Abbott Park, IL, USA). The CLI-gel is dispensed into cassettes connected to an ambulatory programmable pump that delivers the suspension. One cassette supplies 100 ml gel containing 2000 mg levodopa and 500 mg carbidopa that lasts on average 16 h, depending on the individual needs. In some centres, a temporary nasoduodenal tube is used to assess whether the patient responds favorably to continuous levodopa infusion on day 1 to 3. On day 1 or 4 (depending on whether a temporary nasoduodenal tube is used first), a gastroenterologist endoscopically places a PEG tube in the stomach with an extension tube clipped in the jejunum using local anesthetic and sedation with a short acting benzodiazepine. If endoscopic placement fails, a radiologically placed jejunostomy may be performed. The tube is connected to the pump. Thereafter, CLI will immediately be initiated or continued and subsequently adjusted during the hospitalization of, generally, five days. Hereafter, patients will regularly visit the outpatient clinic to further adjust the dose.

### Outcome measures and assessment scales

The primary health economic outcomes of the randomized trial at 12 months follow-up are the costs per unit on the Parkinson’s Disease Questionnaire-39 (PDQ-39) and the costs per Quality Adjusted Life Year (QALY) for the cost-effectiveness and cost-utility analyses respectively. The EuroQol-5D (EQ-5D) will be applied as the utility measure with QALYs calculated as the area under the curve for utility measurements over time after interpolation between successive measurements. Medical and non-medical care costs are evaluated with the Institute for Medical Technology Assessment Medical Consumption Questionnaire (iMCQ) and Institute for Medical Technology Assessment Productivity Cost Questionnaire (iPCQ).

The main clinical outcome is quality of life (PDQ-39). Secondary parameters for the RCT are: (a) laboratory analyses including vitamin B6, B12 and folic acid; (b) PD medication; (c) PD motor symptoms (motor examination in off medication and on medication phase (MDS-UPDRS part III), motor experiences of daily living (MDS-UPDRS part II), Clinical Dyskinesia Rating Scale (CDRS), 3-day motor symptom diary); (d) PD non-motor symptoms (Non-motor symptom checklist, Rotterdam Symptom Checklist); (e) standardized neuropsychological evaluation; (f) psychiatric assessment (Starkstein Apathy Scale, HAM-A, HAM-D, Questionnaire for Impulsive-Compulsive Disorders in Parkinson’s Disease, selected items of the MINI, version 5.0 (see study procedures and randomization for specification of the items), Columbia Suicide Severity Rating Scale); (g) treatment expectations and perceived symptoms (Patient-reported outcome tool for advanced Parkinson’s disease); (h) functional health status (Academic Medical Center Linear Disability Score (ALDS) in off medication and on medication phase, Hoehn and Yahr stage); (i) treatment satisfaction; (j) life satisfaction (Satisfaction With Life Scale); (k) (Serious) Adverse Events, including DBS or CLI device specific failures; (l) number of patients that discontinue DBS or CLI treatment; (m) number of patients that start with an alternative advanced treatment; and (n) caregiver burden. For an overview of all assessments, see Additional file 1: Table S1 for the randomized controlled trial and Additional file 1: Table S2 for the ancillary patient preference observational arms.

### Assessment visits

The initial protocol consists of six specified assessment visits for the Randomized Controlled Trial: at baseline (screening for eligibility for the treatments) and at 1 week, 3 months, 6 months, 9 months, and 12 months after initiation of the study treatment (Visits 1, 2, 3, 4, 5, and 6 respectively). Patients in the ancillary patient preference observational study have three assessment visits: at baseline, and at 9 and 12 months after initiation of the study treatment (Visits 1, 2 and 3). The following baseline characteristics will be assessed in patients in both the RCT and the ancillary patient preference observational study: age, sex, medication, age at onset of Parkinson’s Disease, duration of Parkinson’s Disease, comorbidities and treatment preference. See Additional file 1: Tables S1 and S2 for the assessments performed at the different visits.

#### Extended follow-up

As additional follow-up might provide relevant data on long-term effectiveness, costs, (S)EAs, and cross-overs, an extension of the follow-up duration is intended. After initiation of the study, with additional funding to realize follow-up of an additional two years, visits at 24 months and 36 months will be added (Visits 7 and 8 for the RCT and Visits 4 and 5 for the ancillary patient preference observational study). Patients already participating in the 12-month follow-up will be asked by phone or mail to additionally participate in the 24- and 36-month extension of the study. See Additional file 1: Tables S1 and S2 for the assessments in the extended follow-up.

### Data collection

#### Randomized controlled trial

Visit 1, 6 and 8 are face-to-face meetings with a researcher, for the other visits (Visit 2–5 and 7), patients will be contacted by telephone. Data will be collected in several manners:

- Interview (all visits). In face-to-face and telephone interviews the researcher will read the questions of the questionnaires aloud and fill out the answers of the patient. In Visit 1, 6 and 8, the ALDS will be assessed face-to-face both in on medication phase and in off medication phase; the patient will answer the questions concerning functioning according to the state they are in at that moment. For interviews conducted by telephone, the patient will receive a paper version of the questionnaire in advance, to be able to read along with the researcher during the interview.
- Self-report questionnaires (Visit 1 and 3–8). The patient will fill out questionnaires.
- Physical examination (Visit 1 and 6). The MDS-UPDRS part III in on medication phase and in off medication phase, clinical dyskinesia rating scale and Hoehn and Yahr stage will be assessed by the researcher based on physical examination of the patient.
- Questionnaires filled out by caregiver (Visits 1 and 3–8). A primary care giver selected by the patient will fill out questionnaires. The Starkstein apathy scale will, besides being self-reported by the patient, be filled out as a proxy report stating the primary caregiver’s perspective. Also, the primary caregiver will fill out a questionnaire regarding the burden for the caregiver associated with the patient’s disease.

#### Ancillary patient preference cohort study

- Interview (all visits). In telephone interviews the researcher will read the questions of the questionnaires aloud and fill out the answers of the patient. The patient will receive a paper version of the questionnaire in advance, to be able to read along with the researcher during the interview.
- Assessment by treating physician (Visit 1). The Hoehn and Yahr stage will be provided by the treating physician of the patient.

See Additional file 1: Tables S1 and S2 for specification of the manner of data collection for separate assessments of the RCT and ancillary patient preference cohort study.

#### Data management

Study monitoring and data management will be performed in accordance with the International Conference on Harmonisation – Good Clinical Practice guidelines (ICH-GCP). SAEs will be reported to the Medical Ethics Committee according to national guidelines and will be published with study results. Personal information will be protected according to ICH-GCP and European Privacy Law and will only be available for the coordinating investigators, at screening each patient will be assigned a study ID to maintain anonymity. All data will be entered in a central digitalized database by the investigators, prior to to locking the database. DvP and JD will have access to the final dataset. This is an investigator-initiated study, no other parties will have influence on analysis or publication concerning study data. Trial results will be presented in international peer-reviewed journals and scientific presentations and if they indicate treatment practice should be changed, be incorporated in future guidelines.

### Statistics

#### Sample size calculation

A primarily economic evaluation will be carried out and the sample size has been calculated accordingly. Society’s willingness-to- pay (WTP) per QALY may be indicative of whether or not CLI is affordable compared to DBS. In the Netherlands, a value of €80,000 per additional QALY is considered as a potential but unofficial upper limit of affordability. Considering that CLI may be a less invasive intervention and access to health care is easier to facilitate when two interventions can be provided, a more lenient upper limit for the extra societal costs per additional QALY may be appropriate. Therefore, the sample size calculation is based on a WTP per additional QALY of €120,000 (50% above the potential upper limit in the Netherlands). For comparison, the WTP in the United States is considered $100,000 to $150,000 (≈ €88,000 - €132,000) and in England £20,000 to £50,000 if specific conditions are met (≈ €22,000 - €56,000) [20].

Based on the net health benefit formula suggesting that differences in QALYs between the interventions should be larger than the difference in costs (i.e., €34,174 on average yearly as derived from the expected difference in reimbursements during the life cycle of DBS of five years) divided by the maximum WTP (i.e., €120,000 per QALY) in order for one intervention to be accepted as more efficient than another intervention, CLI treatment should at least outperform DBS treatment by 0.2847 QALY per year. Based on literature data with comparable costs estimates [17], we anticipate standard deviations (SD) for QALYs up to 0.35 and for total costs up to €10,000 (factoring in a 12-month follow-up and non-responders).

To achieve 80% power and given a two-sided significance level of 0.05, for the RCT up to 26 patients per group (52 patients in total) are needed to detect a difference of at least 0.2847 QALY (SD 0.35) for WTP-values up to €120,000 and a worst-case scenario of zero correlation between costs and clinical effect, using a two- group t-test. Accounting for a possible dropout of 20%, we will randomize (26/0.80=) 33 patients per group (66 patients in total).

With this sample size we will also have 80% power to detect a difference in mean PDQ-39 scores (main clinical outcome) between both groups of 10.5 points, assuming the common standard deviation is 15, using a two group t-test with a 0.05 two-sided significance level.

There is no minimal inclusion in the ancillary patient preference observational arms. Based on our clinical experience it is expected that 60% of patients prefers DBS, against 40% preferring CLI. In case 120 patients agree to take part in the ancillary patient preference cohort study (i.e., assuming 72 patients preferring DBS and 48 preferring CLI) and given the same test conditions (common standard deviation of 15 points, 80% power, two group t-test, 0.05 two-sided significance level) we are able to detect:

- a difference in mean PDQ-39 scores of 8.9 points when comparing the effect of DBS treatment of patients who express a preference for that treatment (*n* = 72) with the effect of DBS treatment in the randomized patients (*n* = 33).
- a difference in mean PDQ-39 scores of 9.6 points when comparing the effect of CLI treatment of patients who express a preference for that treatment (*n* = 48) with the effect of CLI treatment in the randomized patients (*n* = 33).

#### Analysis

We will prepare a detailed statistical analysis plan before the database is finalized and locked. However, briefly, the statistical analyses will be based on the intention-to-treat principle. Baseline patient characteristics will be summarized separately for the patients in the four different treatment arms (RCT: DBS and CLI; ancillary patient preference cohort study: DBS and CLI) using means with standard deviations or medians with interquartile ranges for continuous variables (depending on data distributions) and proportions for categorical variables. We will view *p*-values of less than 0.05 as indicative of statistical significance. Statistical uncertainty will be expressed in 95% confidence intervals (CI). We will perform all statistical analyses in the current version of IBM SPSS Statistics for Windows (IBM Corp, Armonk, NY).

#### Randomized controlled trial

The cost-effectiveness and cost utility analyses are conducted from a societal perspective and a time horizon of 12 months. Health care resource use will be gathered with clinical reports forms and with the iMCQ. Unit costing of resources is consistent with existing Dutch guidelines for costing in health care research [21]. Patients are also requested to complete questions on non-reimbursed out-of-pocket expenses by themselves and family members. Data on sick leave from work are gathered with the iPCQ [22, 23] with cost valuation again in accordance with the Dutch costing guideline, thereby applying the friction cost method [24]. Differences in costs and in QALYs between the study groups along with their 95% confidence intervals will be assessed following bootstrapping. Incremental cost-effectiveness and cost-utility analyses too will be performed following bootstrapping and graphically represented as cost-effectiveness planes. Cost-effectiveness acceptability curves, showing the probability of CLI being a cost-effective health care intervention compared with DBS will be reported for levels of societal willingness-to-pay per extra QALY of up to €120,000.

The main clinical outcome – the PDQ-39 follow-up scores at 12 months - will be analyzed using a two-group t-test. Additionally, we will perform multiple linear regression taking into account patients’ PDQ-39 baseline values, the stratifying variable (level of experience of the treatment centre) and (if necessary) for clinically relevant baseline imbalances. The repeated data structure of the PDQ-39 scores will be analyzed using a linear mixed effect model with treatment group as a fixed-effect and an appropriate random effect structure. With regard to the between-group comparisons of the other secondary outcomes we will use the appropriate parametric and non-parametric statistics. The same statistical approaches as described above will be performed when analyzing the complete follow-up data set of *36 months.*

#### Ancillary patient preference cohort study

Baseline characteristics of the randomized and non-randomized group will be compared using the two-group t-test, Mann-Whitney U test and Chi-square test, where appropriate. Both the main clinical outcome and the remaining secondary outcomes in the preference group will be analyzed in accordance with the statistical techniques as used in the RCT-part of the study. With regard to the main clinical outcome at 12 months we will also compare the between-group difference of PDQ-39 scores in DBS-treated patients who were randomized for that treatment or preferred that treatment, using a two-group t-test. The same between-group comparison will be done in the CLI-treated patients. Finally, the impact of the treatment on the PDQ-39 follow-up scores at 12 months in the total group of randomized and non-randomized patients will be analyzed using multiple linear regression, taking into account the PDQ-39 baseline values, significant imbalanced baseline variables and participation in the RCT or preference study.

We consider this RCT, including the ancillary patient preference cohort study, as a negligible risk study. Therefore, no Data Safety Monitoring board is established and no interim analysis is planned.

## Discussion

To compare costs and effectiveness of treatment with DBS and CLI, a prospective, randomized, open label multicentre trial, with two additional ancillary patient preference observational arms will be performed. The results of this trial may answer an important clinical question as it is yet unknown whether the studied therapies are equally effective, whilst CLI treatment probably is considerably more expensive.

In current healthcare provision, optimal allocation of available means becomes more urgent. The results of the INVEST study may decrease unwanted variation in the treatment of advanced PD in current practice. By assessment of the cost-effectiveness an evidence-based choice on the preferred treatment is possible and an unambiguous practice guideline can be developed. Furthermore, valuable information on clinical effect, safety and quality of life will be gathered for both DBS and CLI treatment. The addition of the observational study can increase the potential of the trial results by validating the relatively small randomized study group.

Some facets warrant discussion. First, a blinded design will not be possible, since additional sham procedures are deemed too hazardous and unethical to perform. As the primary health economic outcome and the main clinical outcome are both based on patient-reported perceived quality of life measure (PDQ-39), a blinded endpoint assessment (PROBE-design) is not possible. Secondly, an RCT in combination with ancillary patient preference observational arms (i.e. a patient preference trial or comprehensive cohort study) will be performed. In many countries including the Netherlands, patients and treating neurologists together decide on which of the available therapies to choose and seem to have specific perceptions about the therapies. Consequently, the proportion of patients that is willing to be randomized between the two available treatments may be relatively small and a selection bias may occur. This patient selection may restrict the generalization of the RCT results. Therefore, patients who are not willing to be randomized will be asked to take part in the ancillary patient preference cohort study. These patients are allowed to receive their desired treatment without randomization and will be studied with respect to their baseline characteristics, clinical outcomes and adverse events of the treatment. If the randomized patients resemble the non-randomized patients, the RCT-results reflect a more accurate estimate of the treatment benefits and greater support of its external validity.

In conclusion, the INVEST study is a randomized trial that investigates the cost-effectiveness of PD treatment with DBS and CLI.

### Study details

Amsterdam UMC, University of Amsterdam, Meibergdreef 9, Amsterdam, the Netherlands is the primary sponsor. The study is ongoing at the time of submitting the protocol for publication; the first participant was enrolled on December 19th, 2014 and patients are still being enrolled.

## Supplementary information

**Additional file 1: Table S1.** Assessment schedule randomized controlled trial. **Table S2.** Assessment schedule ancillary patient preference cohort study.

## Data Availability

The most recent version of the full study-protocol can be accessed through the study website www.investamc.nl. Protocol changes will be presented to the Medical Ethics Committee and communicated with local investigators and, if necessary, participating patients and in trial registries.

## Abbreviations

(S)AE: (Severe) Adverse Events
ALDS: Academic Medical Center Linear Disability Score
CAI: Continuous subcutaneous Apomorphine Infusion
CDRS: Clinical Dyskinesia Rating Scale
CI: Confidence Intervals
CLI: Continuous intrajejunal Levodopa Infusion
DBS: Deep Brain Stimulation
EQ-5D: EuroQol-5D
HAM-A: Hamilton Anxiety Rating
HAM-D: Hamilton Depression Rating Scale
iMCQ: Institute for Medical Technology Assessment Medical Consumption Questionnaire
INVEST: Infusion Versus Stimulation
iPCQ: Institute for Medical Technology Assessment Productivity Cost Questionnaire
MDS-UPDRS: Movement Disorder Society - Unified Parkinson’s Disease Rating Scale
MINI: Mini-International Neuropsychiatric Interview
MRI: Magnetic Resonance Imaging
PD: Parkinson’s Disease
PDQ-39: Parkinson’s Disease Questionnaire-39
PEG: Percutaneous Endoscopic Gastrostomy
QALY: Quality Adjusted Life Year
RCT: Randomized Controlled Trial
STN: Subthalamic nucleus
WMO: Medical Research Involving Human Subjects Act (in Dutch: Wet Medisch-wetenschappelijk Onderzoek met Mensen)
WTP: Willingness-to-pay
