# Supplementary material for: Protocol of a randomized open label multicentre trial comparing continuous intrajejunal levodopa infusion with deep brain stimulation in Parkinson’s disease - the INfusion VErsus STimulation (INVEST) study
Source: BMC Neurol. 2020 Jan 31;20:40. doi: 10.1186/s12883-020-1621-y (PMC6995127; doi:10.1186/s12883-020-1621-y)
Supplement: Supplementary file 1 — Additional file 1: Table S1. Assessment schedule randomized controlled trial. Table S2. Assessment schedule ancillary patient preference cohort study. [file 12883_2020_1621_MOESM1_ESM.docx]

| **Table 1: assessment schedule randomized controlled trial** | | | | | | | | | | |
| --- | --- | --- | --- | --- | --- | --- | --- | --- | --- | --- |
| **Outcome** | **Assessment** | **Assessments per visit** | | | | | | | | **Data collection method** |
|  |  | *Visit 1*  *Baseline* | *Visit 2*  *1wk* | *Visit 3*  *3mo* | *Visit 4*  *6mo* | *Visit 5*  *9mo* | *Visit 6*  *12mo* | *Visit 7*  *24mo* | *Visit 8*  *36mo* |  |
| Main outcome measure components |  |  |  |  |  |  |  |  |  |  |
| Quality of life | Parkinson’s Disease Questionnaire-39 | X |  |  |  | X | X | X | X | Self-report questionnaire |
|  | EuroQol-5D | X | X | X | X | X | X | X | X | Self-report questionnaire |
| Medical costs | iMTA Medical Consumption  Questionnaire | X |  | X | X | X | X | X | X | Self-report questionnaire |
| Non-medical care costs | iMTA Productivity Cost Questionnaire | X |  | X | X | X | X | X | X | Self-report questionnaire |
| Secondary outcome measures |  |  |  |  |  |  |  |  |  |  |
| Laboratory analysis^1^ |  | X |  |  |  |  | X |  |  | Medical charts |
| Parkinson’s Disease Medication |  | X | X | X | X | X | X | X | X | Interview |
| Motor symptoms | MDS-UPDRS part II, motor experiences of  daily living | X |  |  |  |  | X |  |  | Self-report questionnaire |
|  | MDS-UPDRS part III, motor score in off  medication and on medication phase | X |  |  |  |  | X |  |  | Physical examination |
|  | Clinical Dyskinesia Rating Scale | X |  |  |  |  | X |  |  | Physical examination |
|  | Motor symptom diary (3 days) | X |  |  |  |  | X | X | X | Self-report questionnaire |
| Non-motor symptoms | Non-motor symptom checklist | X |  |  |  |  | X | X | X | Interview |
|  | Rotterdam Symptom Checklist | X |  |  |  |  | X |  |  | Interview |
| *Table 1 continued* |  |  | | | | | | | |  |
| **Outcome** | **Assessment** | **Assessments per visit** | | | | | | | | **Data collection method** |
|  |  | *Visit 1*  *Baseline* | *Visit 2*  *1wk* | *Visit 3*  *3mo* | *Visit 4*  *6mo* | *Visit 5*  *9mo* | *Visit 6*  *12mo* | *Visit 7*  *24mo* | *Visit 8*  *36mo* |  |
| Standardized  neuropsychological evaluation | See legend^2^ | X |  |  |  |  | X |  | X | Interview |
| Apathy | Starkstein Apathy Scale | X |  |  |  |  | X |  |  | Interview and proxy-report caregiver |
| Anxiety | Hamilton Anxiety Rating Scale | X |  |  |  |  | X |  | X | Interview |
| Depression | Hamilton Depression Rating Scale | X |  |  |  |  | X |  | X | Interview |
| Impulse control disorders | Questionnaire for Impulsive-Compulsive  Disorders in Parkinson’s Disease | X |  |  |  |  | X |  |  | Interview |
| Psychiatric diagnosis | Selected items from Mini-International  Neuropsychiatric Interview^3^ | X |  |  |  |  | X |  | X | Interview |
| Suicide | Columbia Suicide Severity Rating Scale | X |  |  |  |  | X |  | X | Interview |
| Treatment expectations and  perceived symptoms | Patient-Reported Outcome tool for  Advanced Parkinson’s Disease | X |  |  |  |  | X |  | X | Interview |
| Functional health status | ALDS - in off medication phase | X |  |  |  |  | X |  | X | Interview |
|  | ALDS - in on medication phase | X |  |  |  |  | X | X | X | Interview |
|  | Hoehn and Yahr stage | X |  |  |  |  | X | X | X | Physical examination and for visit 7 based on interview |
| Treatment satisfaction | INVEST specific questionnaire |  |  |  |  |  | X | X | X | Interview and  self-report questionnaire |
| Life satisfaction | Satisfaction with Life Scale | X |  |  |  |  | X | X | X | Interview |
| *Table 1 continued* |  |  | | | | | | | |  |
| **Outcome** | **Assessment** | **Assessments per visit** | | | | | | | | **Data collection method** |
|  |  | *Visit 1*  *Baseline* | *Visit 2*  *1wk* | *Visit 3*  *3mo* | *Visit 4*  *6mo* | *Visit 5*  *9mo* | *Visit 6*  *12mo* | *Visit 7*  *24mo* | *Visit 8*  *36mo* |  |
| (Serious) adverse events  including DBS or CLI device  specific failures | INVEST specific questionnaire |  | X | X | X | X | X | X | X | Interview |
| Discontinuing study treatment  and starting other treatment | INVEST specific questionnaire |  |  | X | X | X | X | X | X | Interview |
| Caregiver burden | INVEST specific questionnaire | X |  | X | X | X | X | X | X | Questionnaire filled out by caregiver |

*ALDS, Academic Medical Center Linear Disability Score; AMC, Academic Medical Centre; CT, Computer Tomography; iMTA, institute for Medical Technology Assessments; MDS-UPDRS, Movement Disorder Society - Unified Parkinson’s Disease Rating Scale; MRI, Magnetic Resonance Imaging.*

1. Laboratory analysis: Vitamin B6, Vitamin B12, folic acid, thrombocytes, prothrombin time, activated partial thromboplastin time
2. Standardized neuropsychological assessment: Parkinson’s Disease – Cognitive Rating Scale (PD-CRS), Mattis Dementia Rating Scale (deducted from PD-CRS), Boston Naming Test, Letter Fluency, Wechsler Adult Intelligence Scale IV Similarities, Dutch Adult Reading Test, 15 Word Test, Rivermead Behavioural Memory Test Logical Memory, Trail Making Test, Stroop Colour Word Test, Judgement of Line Orientation, Clock Drawing Test
3. MINI version 5.0 selected items: A: major depressive episode, major depressive disorder, D: (hypo)manic episode, E: panic disorder, F: agoraphobia, G: social anxiety disorder, H: obsessive-compulsive disorder, J: alcohol use disorder, K: substance use disorder, L: psychotic disorder

| **Table 2: assessment schedule ancillary patient preference cohort study** | | | | | | |  |
| --- | --- | --- | --- | --- | --- | --- | --- |
| **Outcome** | **Assessment** | **Assessments per visit** | | | | | **Data collection method** |
|  |  | *Visit 1*  *Baseline* | *Visit 2*  *9mo* | *Visit 3*  *12mo* | *Visit 4*  *24mo* | *Visit 5*  *36 mo* |  |
| Main clinical outcome measure |  |  |  |  |  |  |  |
| Quality of life | Parkinson’s Disease Questionnaire-39 | X |  | X | X | X | Interview |
| Secondary outcome measures |  |  |  |  |  |  |  |
| Parkinson’s Disease Medication |  | X | X | X | X | X | Interview |
| Functional health status | Hoehn and Yahr stage | X |  |  |  |  | Provided by treating  physician |
| Treatment satisfaction | INVEST specific questionnaire |  |  | X | X | X | Interview |
| (Serious) adverse events including DBS or CLI device specific failures | INVEST specific questionnaire |  | X | X | X | X | Interview |
| Discontinuing study treatment and starting other treatment | INVEST specific questionnaire |  | X | X | X | X | Interview |
